# Acute effect on ambulatory blood pressure from aerobic exercise: a randomised cross-over study among female cleaners

**DOI:** 10.1007/s00421-017-3773-z

**Published:** 2017-12-05

**Authors:** Charlotte Lund Rasmussen, Line Nielsen, Marie Linander Henriksen, Karen Søgaard, Peter Krustrup, Andreas Holtermann, Mette Korshøj

**Affiliations:** 1 National Research Centre for the Working Environment, Lersø Parkallé 105, 2100 Copenhagen, Denmark; 2 GoLine, Copenhagen, Denmark; 3 Herlev Gymnasium, Herlev, Denmark; 4 Occupational and Environmental Medicine, University Hospital Odense, Odense, Denmark; 5 Department of Sports Science and Clinical Biomechanics, University of Southern Denmark, Odense, Denmark; 6 Sport and Health Sciences, College of Life and Environmental Sciences, University of Exeter, St. Luke’s Campus, Exeter, UK

**Keywords:** Blood pressure, Aerobic exercise, Worksite intervention, Occupational physical activity, Occupational health, Objective measurements

## Abstract

**Purpose:**

High occupational physical activity (OPA) is shown to increase the risk for elevated blood pressure, cardiovascular diseases and mortality. Conversely, aerobic exercise acutely lowers the blood pressure up to 25 h post exercise. However, it is unknown if this beneficial effect also apply for workers exposed to high levels of OPA. Cleaners constitute a relevant occupational group for this investigation because of a high prevalence of OPA and cardiovascular disease. Accordingly, the objective was to investigate the acute effects on ambulatory blood pressure from a single aerobic exercise session among female cleaners.

**Methods:**

Twenty-two female cleaners were randomised to a cross-over study with a reference and an aerobic exercise session. Differences in 24-h, work hours, leisure time, and sleep ambulatory blood pressure (ABP) were evaluated using repeated measure 2 × 2 mixed-models.

**Results:**

After the aerobic exercise session, the 24-h systolic ambulatory blood pressure was significantly lowered by 2.4 mmHg (*p* < 0.01) compared to the reference session. The 24-h diastolic ABP was unaltered. During work hours, a lowered systolic ABP of 2.2 mmHg (*p* = 0.02) and a higher diastolic ABP of 1.5 mmHg (*p* = 0.03) were found after the aerobic exercise session. During leisure time, the systolic ABP was lowered by 1.7 mmHg (*p* = 0.04) and the diastolic ABP was unaltered. During sleep, the systolic and diastolic ABP was unaltered.

**Conclusion:**

A single aerobic exercise session lowered 24-h systolic ABP of 2.4 mmHg. Thus, an aerobic exercise session seems to be beneficial for lowering the risk of hypertension among cleaners.

## Introduction

Hypertension markedly increases the risk of cardiovascular disease and contributes annually to approximately 7.6 million deaths worldwide (Chobanian et al. 2003; Antikainen et al. 2006; Lawes et al. 2008). Physical activity, and especially aerobic exercise, is a recommended non-pharmacological treatment for hypertension (Mach et al. 2005). This recommendation is mostly based on sport and exercise studies, showing aerobic exercise to lower blood pressure, enhance cardiorespiratory fitness, and preserve cardiovascular health (Whelton et al. 2002; Crivaldo Gomes Cardoso 2010).

Conversely, evidence is emerging for workers with high occupational physical activity having an increased risk of cardiovascular diseases (Holtermann et al. 2012). This increased risk could be caused by individual factors such as smoking status, dietary habits, obesity and lower socioeconomic status (Joseph et al. 2017). In addition, it could be a result of high levels of occupational physical activity (Holtermann et al. 2011). For example, cleaners being exposed to high levels of occupational physical activity have an increased risk of hypertension, cardiovascular disease and mortality (Sjögren et al. 2003; Søgaard et al. 2006). Cleaning requires repetitive movements of the arms combined with high levels of both static and whole-body dynamic muscular strain (Sjögren et al. 2003; Søgaard et al. 2006; Korshøj et al. 2013). This strenuous occupational physical activity increases heart rate (Aastrand et al. 1968; Sjögren et al. 2003). In particular, workers with a low cardiorespiratory fitness level will experience an elevated heart rate (Søgaard et al. 2006). Acutely, the elevated heart rate leads to a higher aerobic workload and subsequently an increase in blood pressure (Korshøj et al. 2016a). On the long term, an elevated heart rate and blood pressure from high occupational physical activity will induce excessive sheer stress on the atrial wall, increasing the risk of inflammation and hypertension (Glagov et al. 1988; Chobanian et al. 2003).

To our knowledge, no studies have investigated the acute effect of a single aerobic exercise session on blood pressure among workers with high levels of occupational physical activity, such as cleaners. Two studies examined the long-term effects on cardiorespiratory fitness and blood pressure from 30-min of aerobic exercise twice a week for 4 months among cleaners in Denmark (Korshøj et al. 2015, 2017). The authors found improved cardiorespiratory fitness, but also an increased systolic blood pressure (Korshøj et al. 2015, 2017). To understand a part of the cardiovascular mechanism explaining these findings, additional research was needed.

Thus, the aim of this study was to investigate the acute effect of a single aerobic exercise session on ambulatory blood pressure among cleaners.

## Methods

### Ethical approval

The study was approved by the Ethics Committee for the regional capital in Denmark (journal number H-2-2011-116) and conducted according to the 1964 Helsinki declaration and its later amendments.

### Study design

The study was a controlled, randomised cross-over trial. Participants were randomised to either begin with 24-h reference diurnal measurement (REF) or an aerobic exercise session followed by 24-h diurnal measurement (AES). After a washout period of 1 week, the participants would cross over and undergo the opposite of the first week.

### Recruitment

An aircraft cleaning company, located in Copenhagen, Denmark, was recruited by direct contact. All cleaners were invited to an information meeting where the project was presented. All attending cleaners received information leaflets and a screening questionnaire. Consent to participate was obtained via the screening questionnaire.

Cleaners were eligible if they worked more than 20 h per week; were aged between 18 and 65 years old; and worked fixed day or evening shifts or two-shift schedules (day/evening). Informed consent was obtained from all individual participants included in the study. Exclusion criteria were pregnancy and fever on the day of testing. Participants with severe hypertension (≥ 160/≥100 mmHg) at baseline; angina pectoris; daily intake of prescribed cardio- or respiratory medicine; and/or pacemakers were excluded from the aerobic exercise session.

### Data collection

Data were collected in November 2013. Data collection included a baseline test, baseline health measurements, two 24-h diurnal measurements (REF and AES) and a 30-min aerobic exercise session.

The baseline test consisted of a structured interview, questionnaire, and a health check. During the interview, data were collected on medical history and lifestyle. Information on work hours and seniority was collected by questionnaire. Baseline health measurements included anthropometrics and resting systolic and diastolic blood pressures which were measured three consecutive times using an Omron blood pressure monitor, Model M6 Comfort. During measuring, the participant was asked to relax and sit in an upright position (Omron healthcare UK LTD., England). The lowest pair of measured blood pressures was registered as the consultation blood pressure. Participants with hypertension (≥ 140/≥90 mmHg) were informed and advised to consult their physician for further examination.

Both REF and AES were conducted on days including work and consisted of measurements of physical activity, ambulatory blood pressure (ABP) and heart rate. The participants were asked to wear all monitors continually for 24-h. Moreover, the participants were asked to keep a log of their time at work, when they went to bed and got up in the morning, and any periods spent without monitors during the 24 h. During the period of the 24-h diurnal measurements, the participants were instructed to pursue their ordinary lives. Physical activity was measured using Actigraph GT3X+ accelerometers (ActiGraph LLC, Pensacola, FL, USA) fixed just below the right crista iliaca and halfway between the crista iliaca and the patella at the medical front of the right thigh (Skotte et al. 2014). The accelerometers were fixed using double-sided adhesive tape (3 M, Hair-Set, St. Paul, Minnesota, USA), Fixomull (Fixomull BSN medical GmbH, Hamburg, Germany) and a waterproof film (OpSite Flexifix, Smith & Nephew, London, England).

Data were downloaded using Actilife Software version 5.10.0 (“Software, Actilife”) and further analysed using the custom-made MATLAB programme Acti4 (Skotte et al. 2014). The programme has been validated and shown to identify the following physical activities with high sensitivity and specificity based on the accelerometer data: standing, sitting, slow and fast walking, cycling, and running (Korshøj et al. 2014; Stemland et al. 2015).

Ambulatory blood pressure was measured with a Spacelabs monitor (Model 90217, Spacelabs Medical Inc., Redmond, WA, USA) by oscillometry. This monitor is validated for measurement of blood pressure in the field (Baumgart and Kamp 1998). The monitor was mounted on the non-dominant upper arm with a tube connecting the sampler to the cuff. The measurement frequency was once every 20 min during waking hours (from 5 a.m. to 10 p.m.) and once every 40 min during night time (from 10 p.m. to 5 a.m.) (Clays et al. 2012). The participants were asked to remain quiet and keep their arm at rest while the measurements took place. If a measurement failed, the monitor would automatically repeat the measurement after 3 min. Moreover, the participants were instructed in how to handle and remove the Spacelabs monitor during showering.

Heart rate was measured using Actiheart (Barreira et al. 2009) (CamNtech LTD., Cambridge, England) mounted with Ag/AgCl pre-gelled electrodes (Ambu blue sensor VL-00-S/25, Ambu A/S, Ballerup, Denmark) at the validated position at the sternum with the wire horizontal to the left lateral intercostal (Brage et al. 2005). Actiheart was initialised using the height, weight, age and sex of the participant. Data were downloaded in the Actiheart software (version 4.0.100) (CamNtech 2016) and analysed in the Acti4 software (Skotte et al. 2014).

Each participant performed the 30-min aerobic exercise session once during work hours, at the beginning of the work shift. The session was supervised and performed on a bicycle ergometer (Monark Ergomedic 874E, HaB International Ltd., Southam, Warwickshire, England). During the aerobic exercise session, heart rate was monitored via Actiheart and a finger oximeter (OxiMax Pulse Oximetry System, Nellcor, N-65, COVIDIEN, USA). The session began with an Aastrand submaximal one-point fitness test to determine the participants’ cardiorespiratory fitness (Åstrand and Ryhming 1954). The test lasted 10 min at most after which the aerobic exercise session would begin. The aerobic exercise session consisted of 13 blocks lasting 30–60 s and alternating between work and active breaks. The target intensity of the aerobic exercise was ≥ 80% of maximal heart rate (HR_max_) for 11 min in total, ≥ 70% of HR_max_ for 6 min in total, ≥ 60% of HR_max_ for 9 min in total and ≥ 55% of HR_max_ for 4 min in total. The aim was for the total 30-min aerobic exercise session to be performed with an average intensity of ≥ 70% of HR_max_.

### Data analysis

Information on current smoking status was dichotomised, corresponding to the participant being “smoker/non-smoker”. Body mass index (BMI) was calculated using the equation BMI = [body weight (kg)/body height (m^2^)] (Canoy 2008). Consultation blood pressure was defined based on the measurement with the lowest diastolic and systolic values at the baseline health measurement. Percent leisure time spent on sedentary behaviour (sitting/lying) and percent work hours spent on feet was measured by the Actigraphs.

Heart rate data were filtered and physiological outliers (< 30 and > 220 beats/min) excluded. Only non-outliers and heart rate measurements with < 50% beat error were included in the statistical analysis (Skotte et al. 2014). Heart rate reserve (HRR) was defined as the difference between estimated HR_max_ and sleeping heart rate (SHR) (HRR = HR_max_ – SHR) (Karvonen et al. 1957). HR_max_ was determined by age by the Tanaka equation (Fox et al. 1971). SHR was defined as the tenth lowest recorded heart rate value during sleep (Brage et al. 2004). The mean relative aerobic workload was calculated as percent of estimated HRR (%HRR = mean heart rate during work/HRR × 100%). The %HRR is well documented to provide a measure of the physiological cardiorespiratory strain on the body depending on the work demands and cardiorespiratory fitness of the participant (Ilmarinen 1992).

Based on the participants’ logs, all 24-h data were sorted into work, leisure and sleep. All measurements of ABP were visually checked for physiological outliers (systolic blood pressure < 80 and > 240 mmHg, diastolic blood pressure < 50 and > 130 mmHg). ABP measurements for an participant were included in the analysis when a minimum of 25% of planned measurements were complete, corresponding to a total of 15 measurements: five during work, eight during leisure and three during sleep (Baumgart and Kamp 1998).

### Statistical analysis

All analyses were performed according to the intention-to-treat principle (Detry and Lewis 2014). Missing values were not imputed. The difference between ABP as measured during REF and AES was estimated using a repeated-measures 2 × 2 mixed-model analysis. The fixed factor was randomised group (REF and AES). Each participant was entered in the model as a random effect. Moreover, baseline value of the respective outcome (consultation blood pressure) was entered as random effect.

Two stratified analysis were performed using the same model stratified by %HRR during work hours (≥/< 30% of HRR) and by smoking status (smoker/non-smoker).

Statistical estimates of the outcome are reported as mean differences in ABP during REF and AES, standard errors of the mean, 95% confidence intervals and *p* values. A significance level of ≤ 0.5 was used. All statistical analyses were conducted using the SAS statistical software for Windows (version 9.3) (“SAS”).

### Data availability

The datasets analysed during the current study are not publicly available due to difficulties with ensuring anonymity because of the small study population. However, data are available from the corresponding author on reasonable request.

## Results

### Flow

**Fig. 1:**
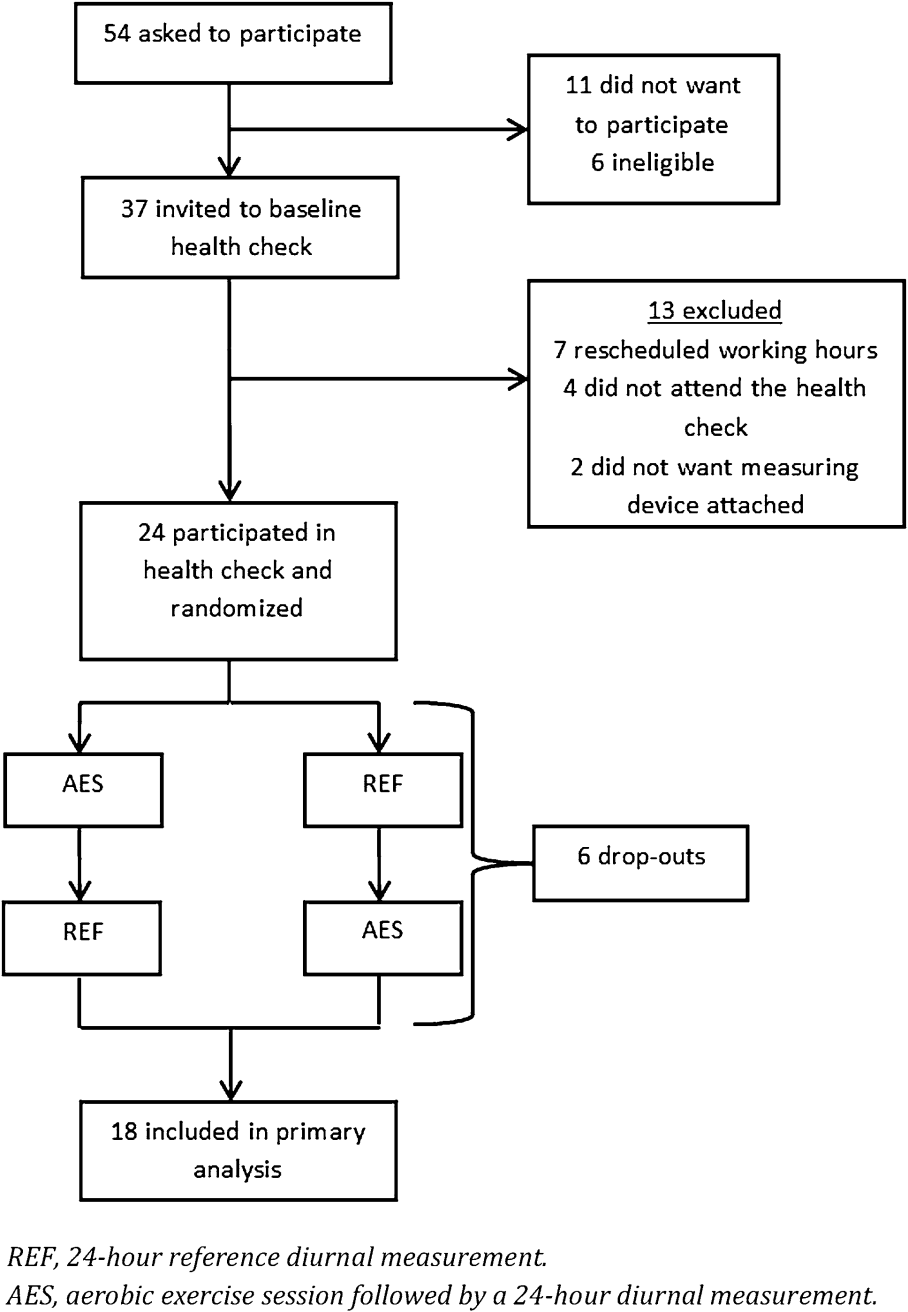
Flow chart of the study population

Figure 1 shows the trial profile. A total of 54 cleaners were invited to participate in the study, of which 43 participated. Six cleaners were excluded as they did not meet the work hour criterion (at least 20 work hours a week). Thus, 37 were invited to the baseline health measurement. Of the 37 invited cleaners, 13 were excluded because they did not want to wear the Actiheart, did not attend the health measurement, or had changes in their work schedule. A total of 24 cleaners were randomised to begin with either 24-h reference diurnal measurement (REF) or an aerobic exercise session followed by 24-h diurnal measurement (AES). After 1 week, the participants would cross over and undergo the opposite of the first week. Six cleaners dropped out after the randomisation, resulting in 18 participants completing both the baseline health measurement, 24-h diurnal REF and AES measurements. The 18 participants performed the 30-min aerobic exercise session with an average intensity of 81.4% of HR_max_ [SD = 5.2 (data not shown)].

### Descriptive characteristics

Baseline characteristics of the study population included in the primary analysis are presented in Table 1. All participants were normotensive with consultation blood pressures below 140/90 mmHg. Mean aerobic workload during cleaning was 30.2% HRR (SD = 5.4) and 39% worked at an aerobic workload ≥ 30%HRR. The participants spent on average 47.0% (SD = 9.6) of their work hours on their feet and 54.9% (SD = 15.4) of their leisure time sedentary. Of the 18 participants, nine (50%) were smokers.

**Table 1:** Descriptive characteristics of 18 participants included in the primary analysis

Variable	%	Mean	SD	*n*
Age (years)		44.7	7.7	18
Height (m)		1.7	6.9	18
Weight (kg)		64.4	9.7	18
BMI (kg/m^2^)		22.5	2.7	18
BMI ≥ 25 kg/m^2^	16.7			3
Cardiorespiratory fitness (mlO_2_/min/kg)		33.1	8.2	16
Job seniority in cleaning (years)		14.6	7.3	17
Working hours per week		36.1	2.6	18
Consultation systolic blood pressure (mmHg)		113.7	8.2	18
Consultation diastolic blood pressure (mmHg)		76.6	6.4	18
Aerobic workload during cleaning (% HRR)		30.2	5.4	17
Sedentary leisure time (% of leisure time)		54.9	15.4	17
Working on feet (% of work time)		47.0	9.6	18
Aerobic workload ≥ 30% HRR	38.9			7
Current smoker	50.0			9
Ethnicity (% from Western countries)	94.4			17

*SD* standard deviation, *n* number, *HRR*, heart rate reserve

### Primary analysis

Group mean of ABP during the REF and the AES and corresponding differences in 24-h ABP are shown in Table 2. A significant difference in systolic 24-h ABP of − 2.4 mmHg (*p* < 0.01) was seen. During work hours, the results showed a significant difference in systolic ABP of − 2.2 mmHg (*p* = 0.02) and in diastolic ABP of 1.5 mmHg (*p* = 0.03). During leisure, a significant difference in systolic ABP of − 1.7 mmHg (*p* = 0.04) was found. During sleep, no differences were found in either systolic or diastolic ABP (Table 2).

**Table 2:** Group means pre (REF) and post (AES) intervention and differences in pre- to post intervention changes of ambulatory blood pressure (ABP)

ABP (mmHg)	REF	AES	Δ	SE	95% CI	*p*
	Mean	Mean				
24-h						
Systolic	122.0	119.6	− 2.4	0.6	− 3.5; − 1.3	< 0.01**
Diastolic	76.6	76.6	− 0.04	0.4	− 0.9; 0.8	0.92
Work hours						
Systolic	125.3	123.1	− 2.2	0.9	− 3.9; − 0.3	0.02*
Diastolic	77.9	79.5	1.5	0.7	0.2; 2.8	0.03*
Leisure time						
Systolic	121.9	120.2	− 1.7	0.8	− 3.4; − 0.1	0.04*
Diastolic	77.5	77.4*	− 0.2	0.6	− 1.4; 1.1	0.81
Sleep time						
Systolic	102.6	103.6	1.1	1.2	− 1.1; 3.3	0.34
Diastolic	61.8	62.7	0.9	0.8	− 0.8; 2.6	0.28

Model adjusted for consultation blood pressure

*REF* 24-h reference diurnal measurement, *AES* aerobic exercise session followed by a 24-h diurnal measurement, *ABP* ambulatory blood pressure, ***Δ*** delta, *SE* standard error, *CI* confidence interval

*p < 0.05, **p < 0.01

### Analysis stratified by level of aerobic workload

Table 3 shows the results of the secondary analysis stratified by aerobic workload (≥/< 30% of HRR during work). Among the participants exposed to a high aerobic workload (*n* = 7), the differences in ABP between AES and REF over 24-h were − 4.3 mmHg systolic (*p* < 0.01); during work hours − 3.7 mmHg systolic (*p* = 0.01) and 2.3 mmHg diastolic (*p* = 0.03); and during leisure time − 5.2 mmHg systolic (*p* < 0.01) and − 1.7 mmHg diastolic (*p* = 0.12). During sleep, no difference in ABP was observed (Table 3). Among those exposed to a low aerobic workload (*n* = 10), only systolic ABP during leisure time reached a significant difference between AES and REF of 2.9 mmHg (*p* = 0.01).

**Table 3:** Group means pre (REF) and post (AES) intervention and differences in pre to post intervention changes of ambulatory blood pressure (ABP) stratified by level of aerobic workload (< 30% HRR, ≥ 30% HRR)

ABP (mmHg)	REF	AES	Above 30% HRR during work					REF	AES	Below 30% HRR during work				
	Mean	Mean	Δ	SE	95% CI	*p*	*n*	Mean	Mean	Δ	SE	95% CI	*p*	*n*
24-h														
Systolic	121.1	116.7	− 4.3	0.9	− 6.2; − 2.4	< 0.01**	7	122.8	120.2	− 0.02	0.7	− 1.4; 1.4	0.98	10
Diastolic	76.7	76.6	0.1	0.7	− 1.3; 1.4	0.91	7	76.9	75.2	0.1	0.5	− 0.9; 1.5	0.86	10
Work hours														
Systolic	123.4	120.7	− 3.7	1.5	− 6.4; − 0.8	0.01*	7	125.7	125.0	− 0.2	1.3	− 2.7; 2.3	0.87	10
Diastolic	77.4	80.5	2.3	1.0	− 0.2; 4.3	0.03*	7	77.3	78.1	0.9	0.9	− 0.9; 2.7	0.32	10
Leisure time														
Systolic	123.2	118.0	− 5.2	1.4	− 7.9; − 2.5	< 0.01**	7	119.9	124.8	2.9	1.1	0.7; 4.9	0.01*	10
Diastolic	78.4	76.6	− 1.7	1.1	− 3.8; 0.4	0.12	7	75.8	78.9	1.1	0.8	− 0.5; 2.7	0.18	10
Sleep time														
Systolic	93.5	97.1	0.7	1.4	− 2.1; 3.5	0.62	7	104.7	106.7	0.7	1.1	− 1.4; 2.8	0.51	10
Diastolic	58.7	60.5	0.7	1.1	− 1.4; 2.8	0.51	7	62.2	64.4	1.2	1.3	− 1.3; 3.8	0.34	10

Models adjusted for consultation blood pressure

*REF* 24-h reference diurnal measurement, *AES* aerobic exercise session followed by a 24-h diurnal measurement, *ABP* ambulatory blood pressure, *Δ* delta, *SE* standard error, *CI* confidence interval

*p < 0.05, **p < 0.01

### Analysis stratified by smoking status

When stratifying by smoking status (smoker/non-smoker), the beneficial acute effect on ABP of the aerobic exercise session was pronounced among non-smokers (results not shown). In contrast, no beneficial effects were seen among smokers (results not shown).

## Discussion

The aim of this study was to investigate the acute effect of a single aerobic exercise session on ABP on the following 24-h among cleaners. The primary analysis showed a lowered 24-h systolic ABP following the aerobic exercise session, compared with the reference measurement (Table 2). Previously, a single aerobic exercise session has been found to acutely decrease systolic and diastolic ABP by 2–12 mmHg post exercise in both normotensive and hypertensive individuals in populations of unspecified occupation (Whelton et al. 2002; Crivaldo Gomes Cardoso 2010). Corresponding results were found in the present study in a population of cleaners with high levels of occupational physical activity.

Modest differences in systolic ABP between REF and AES were observed during leisure time and sleep within the 24-h (Table 2). During work hours, an overall beneficial effect of the aerobic exercise session of 2.4 mmHg lowered systolic ABP was found. This difference might seem small, especially when applied to normotensive persons. However, an overall decrease in systolic ABP of 2 mmHg is expected to reduce the risk of coronary heart disease by 4%, of stroke by 6%, and of cardiovascular mortality by 7% if applied to the general population (Whelton et al. 2002; Lewington et al. 2002; Chobanian et al. 2003). Moreover, the lowered systolic ABP during work hours is particularly promising given that cleaners are expected to experience elevated blood pressure during work hours as a result of high aerobic workloads (Korshøj et al. 2016a). We also found a significantly 1.5 mmHg higher diastolic ABP during work hours (Table 2). However, when applied to the general population, an increase of 1.5 mmHg in diastolic ABP is not expected to increase cardiovascular hazards and therefore not considered clinically significant (Brguljan-Hitij et al. 2014).

When stratifying according to level of aerobic workload at baseline (≥/< 30% of HRR), we found contrasting differences in ABP between REF and AES in the two groups. Among cleaners with low aerobic workload, a higher systolic ABP during leisure time was seen (Table 3). This increase could raise concerns about the intended cardiovascular benefits of initiating worksite aerobic exercise sessions. However, the overall 24-h ABP was unchanged in this subgroup. Thus, no overall harmful effect of clinical significance ought to be expected. Nevertheless, this result should to be investigated in futures studies in comparable study populations. Among cleaners with high aerobic workload, we found lowered systolic ABP during the 24-h, work hours, and leisure time (Table 3). As a high level of aerobic workload increases the risk of elevated blood pressure (Korshøj et al. 2016a), these results suggest an acute beneficial cardiovascular response in a high-risk population of workers. Notably, a 4-month intervention study that conducted similar aerobic exercise intervention to a population of cleaners, found an increased ABP at follow-up (Korshøj et al. 2015). Thus, a long-term exposure to aerobic exercise could lead to adverse effect on blood pressure among workers with high levels of occupational physical activity.

### Practical implications

There is a need to understand how to reduce cardiovascular risks in occupational groups with high levels of occupational physical activity. Cleaners constitute a high-risk group because of their exposure to high levels of occupational physical activity (Sjögren et al. 2003; Korshøj et al. 2016a).

In this study, we found significantly lowered systolic ABP following a single 30-min aerobic exercise session, indicating beneficial effects on ABP in this group of cleaners. This suggests that the previously found beneficial acute effect of aerobic exercise also applies for workers with high levels of occupational physical activity.

### Strengths and limitations

The major strengths of this study are the validated and precise 24-h ABP measurements and the diurnal heart rate and physical activity measurements. Such objective measurements decrease the risk of subjective recall bias and bias from differential misclassification. Furthermore, ABP has been shown to be superior to clinical blood pressure measurements in predicting risk of cardiovascular diseases (Perloff et al. 1983). The cross-over design is a methodological strength, the benefits of which include direct comparison of the interventions under investigation among the same participants, thereby eliminating between-subject variability and minimising the risk of bias (Rothman et al. 2008). Moreover, the use of a mixed-model enabled analysis with missing data, thereby reducing the possible bias associated with multiple imputations (Twisk et al. 2013). Finally, the cleaners in this study were all normotensive with a mean baseline blood pressure of 114/77 mmHg.

The generalizability of the current study is limited because of the sample of cleaners working at an aircraft cleaning company in Copenhagen, Denmark. Compared to other populations of cleaners (Sjögren et al. 2003; Søgaard et al. 2006; Korshøj et al. 2016b), our study population had lower BMI and a higher baseline cardiorespiratory fitness. Moreover, the generalizability could be limited because of the female study population. However, studies show no indication of sex difference in post-exercise hypotension. Accordingly, the observed lowered blood pressure is most likely not depending on sex (Queiroz et al. 2013). Finally, the secondary stratified analysis by aerobic workload was based on very small strata, thereby limiting the robustness of the results.

## Conclusion

In this cross-over study, we found a lowered 24-h systolic ABP after a single 30-min aerobic exercise session. Although the magnitude of the reduction in ABP might appear modest, previous literature shows changes of 2 mmHg to be of clinical importance. Thus, our results indicate beneficial acute effect on ABP. The beneficial acute effect on ABP was especially noticeable among cleaners with high aerobic workload. As far as we know, this is the first study to evaluate the acute ABP effects of a single aerobic exercise session in workers with high levels of occupational physical activity. Thus, this study provides new knowledge regarding the acute ABP effects of aerobic exercise in a high-risk population. However, future studies are needed to understand and verify the identified acute ABP effect of a single aerobic exercise session in workers with high levels of occupational physical activity. We recommend future studies evaluating blood pressure effects to monitor 24-h ABP and to consider the level of occupational physical activity of the study population.

## Abbreviations

ABP: Ambulatory blood pressure
AES: Aerobic exercise session followed by 24-h diurnal measurement
BMI: Body mass index
HR_max_: Maximal heart rate
HRR: Heart rate reserve
REF: 24-h reference diurnal measurement
SHR: Sleeping heart rate
